# Genetic association of *PRKCD* and *CARD9* polymorphisms with Vogt–Koyanagi–Harada disease in the Chinese Han population

**DOI:** 10.1186/s40246-023-00459-7

**Published:** 2023-02-13

**Authors:** Chunya Zhou, Shiya Cai, Yuhong Xie, Zhen Zeng, Jun Zhang, Guannan Su, Qiuying Wu, Xingsheng Ye, Qingfeng Cao, Peizeng Yang, Jianmin Hu

**Affiliations:** 1 Department of Ophthalmology, The Second Affiliated Hospital of Fujian Medical University, Engineering Research Center of Assistive Technology for Visual Impairment, Fujian Province University, Quanzhou, 362000 People’s Republic of China; 2 Department of Ophthalmology and Optometry, The School of Medical Technology and Engineering, Fujian Medical University, Jiaotong Road 88, Fuzhou, 350004 People’s Republic of China; 3 The First Affiliated Hospital of Chongqing Medical University, Chongqing Key Laboratory of Ophthalmology and Chongqing Eye Institute, Youyi Road 1, Chongqing, 400016 China

**Keywords:** Vogt–Koyanagi–Harada disease, Genetic susceptibility, Single-nucleotide polymorphism, Caspase recruitment domain family member 9, Protein kinase C delta

## Abstract

**Background:**

Protein kinase C delta (*PRKCD*) and caspase recruitment domain family member 9 (*CARD9*) are genes involved in B and T cell activation, and cytokine production, which are vital mechanisms underlying autoimmune disease development. This study aimed to explore the association of the *PRKCD* and *CARD9* genes with Vogt–Koyanagi–Harada disease (VKH) disease. The case–control study was performed to in 912 patients with VKH and 878 normal controls. MassARRAY system, SHEsis online platform, real-time PCR, and enzyme-linked immunosorbent assay were used to detect genotyping, haplotyping, mRNA expression, and cytokine levels, respectively.

**Results:**

We found that rs74437127 C allele of *PRKCD*, rs3812555 CC genotype, and C allele of *CARD9* were associated with increased susceptibility of VKH (Pc = 0.020, OR = 1.624; Pc = 2.04 × 10^–5^, OR = 1.810; Pc = 2.76 × 10^–5^, OR = 1.698, respectively). However, the rs74437127 T allele, and rs3812555 TC genotype and T allele were linked with decreased susceptibility to VKH (Pc = 0.020, OR = 0.616; Pc = 7.85 × 10^–5^, OR = 0.559; Pc = 2.76 × 10^–5^, OR = 0.589, respectively). *PRKCD* ATG and *CARD9* GCTTA haplotypes decreased susceptibility to VKH (Pc = 3.11 × 10^–3^, OR = 0.594; Pc = 5.00 × 10^–3^, OR = 0.639, respectively). Functional studies on rs3812555 genotyped individuals revealed that CC carriers had significantly higher *CARD9* mRNA expression and tumour necrosis factor-α production than TC/TT carriers (*P* = 1.00 × 10^–4^; *P* = 2.00 × 10^–3^, respectively).

**Conclusions:**

We found an association between *PRKCD* rs74437127 and *CARD9* rs3812555 polymorphisms and VKH susceptibility and revealed that the increased susceptibility of rs3812555 for VKH may be mediated by regulating *CARD9* gene expression and the production of pro-inflammatory cytokines, such as TNF-α.

**Supplementary Information:**

The online version contains supplementary material available at 10.1186/s40246-023-00459-7.

## Background

Vogt–Koyanagi–Harada (VKH) disease is a recurrent systemic autoimmune disease characterised by bilateral granulomatous uveitis, which is usually accompanied by central nervous system signs, vitiligo, and skin and hair abnormalities[1]. VKH disease is one of the most prevalent subtypes of uveitis in China, timely and appropriate treatment can prevent irreversible visual impairment and blindness in patients with VKH [2]. The aetiopathogenesis of this disease remains unclear, but it is generally considered as T cell-mediated autoimmune disease caused by abnormal responses of genetically susceptible individuals to environmental triggers [3, 4]. Earlier studies have shown that several immune response-related genes are associated with VKH susceptibility in the Chinese Han population [4–6]. Understanding the genetic mechanism underlying VKH disease may provide a basis for identifying new targets and developing new therapeutic strategies for VKH disease [7].

Impaired immune tolerance and enhanced immune activation are deemed important risk factors for the development of autoimmune diseases [8]. The *PRKCD* gene, encoding protein kinase C delta (PKCδ), plays a critical role in the survival, proliferation, and apoptosis of various types of cells, including lymphocytes [9]. PKCδ, a signalling kinase that regulates many cellular responses, serves as a critical regulator of peripheral B cell development and immune homeostasis [10, 11], and plays a negative regulatory role in T cell proliferation and T cell receptor (TCR)/CD3-mediated interleukin (IL)-2 production [12]. In addition, *PRKCD* polymorphisms are associated with increased risk of both systemic lupus erythematosus (SLE) [13] and Crohn’s disease (CD) [14]. Caspase recruitment domain family member 9 (CARD9) not only transmits various signals from natural immune system pattern recognition receptors [15], but also is highly expressed in antigen-presenting cells (APCs) [16] and provides a signal for initiating an adaptive immune response. As an important immune adaptor protein, CARD9 is a bridge linking the natural immune responses elicited by pathogens and acquired immune responses associated with autoimmune diseases [15]. Moreover, *CARD9* polymorphisms predispose individuals to autoimmune diseases, such as ankylosing spondylitis (AS) [17], rheumatoid arthritis (RA) [18], inflammatory bowel disease (IBD) [19], and IgA nephropathy [20].

The role of *PRKCD* and *CARD9* genetic variations in predisposition to VKH disease has not been studied yet. Therefore, this study aimed to explore the association of the *PRKCD* and *CARD9* genes with VKH disease.

## Results

### Clinical features of the study population

The clinical feature distribution of the 912 enrolled patients with VKH is shown in Table 1. The clinical features were as follows: uveitis (100%), sunset glow fundus (48.7%), headache (49.2%), tinnitus (45.0%), vitiligo (11.4%), and alopecia (31.4%). The age and sex distributions of the 878 normal controls have also been provided in Table 1.

**Table 1:** Clinical features of participants in this study

Clinical features	VKH group (%)	Control group (%)
Patients with VKH	912	878
Mean age ± SD	40.53 ± 11.50	40.13 ± 10.47
Male	461 (50.5%)	415 (47.3%)
Female	451 (49.5%)	463 (52.7%)
Uveitis	912 (100%)	–
Sunset glow fundus	444 (48.7%)	–
Headache	449 (49.2%)	–
Tinnitus	410 (45.0%)	–
Vitiligo	104 (11.4%)	–
Alopecia	286 (31.4%)	–

VKH Group, VKH patients; Control group, healthy controls

### Genotyping results for the SNPs tested

Thirteen tag SNPs of the *PRKCD* and *CARD9* genes were genotyped to compare the genotype and allele frequencies in the 912 patients with VKH and 878 normal controls. Our study achieved 90.5% statistical power in detecting genetic association signals of each tag SNP (Additional file 1: Table S2 and Fig. S1). Thirteen tag SNPs had a call rate above 90% and did not deviate from the HWE (P ≥ 0.05; Additional file 1: Table S3).

Patients with VKH exhibited a significantly higher frequency of C allele and lower frequency of T allele of *PRKCD* rs74437127 than the controls (Pc = 0.020, OR = 1.624, 95% CI = 1.200–2.199; Pc = 0.020, OR = 0.616, 95% CI = 0.455–0.833, respectively). In the case of *CARD9* rs3812555, patients with VKH exhibited significantly higher frequency of CC genotype and C allele, and lower frequency of TC genotype and T allele than the controls (Pc = 2.04 × 10^–5^, OR = 1.810, 95% CI = 1.418–2.311; Pc = 2.76 × 10^–5^, OR = 1.698, 95% CI = 1.362–2.118; Pc = 7.85 × 10^–5^, OR = 0.559, 95% CI = 0.434–0.721; Pc = 2.76 × 10^–5^, OR = 0.589, 95% CI = 0.472–0.734, respectively; Table 2). The other 11 tag SNPs examined did not show any association with VKH disease (Pc > 0.05; Additional file 1:Table S4).

**Table 2:** Association of *PRKCD* rs74437127 and *CARD9* rs3812555 with VKH diseases

Gene	SNPs	Genotype allele	VKH n (%)	Control n (%)	P value	Pc value	OR(95% CI)
*PRKCD*	rs74437127	TT	0 (0.000)	7 (0.008)	0.007*	NS	–
		TC	73 (0.080)	97 (0.111)	0.026	NS	0.698 (0.507–0.960)
		CC	839 (0.920)	771 (0.881)	0.006	NS	1.550 (1.132–2.124)
		T	73 (0.040)	111 (0.063)	0.001	0.020	0.616 (0.455–0.833)
		C	1751 (0.960)	1639 (0.937)	0.001	0.020	1.624 (1.200–2.199)
*CARD9*	rs3812555	CC	695 (0.840)	602 (0.744)	1.57 × 10^–6^	2.04 × 10^–5^	1.810 (1.418–2.311)
		TC	119 (0.144)	187 (0.231)	6.04 × 10^–6^	7.85 × 10^–5^	0.559 (0.434–0.721)
		TT	13 (0.016)	20 (0.025)	0.221*	NS	0.630 (0.311–1.275)
		C	1509 (0.912)	1391 (0.860)	2.12 × 10^–6^	2.76 × 10^–5^	1.698 (1.362–2.118)
		T	145 (0.088)	227 (0.140)	2.12 × 10^–6^	2.76 × 10^–5^	0.589 (0.472–0.734)

VKH, VKH disease; OR, odds ratio; 95% CI, 95% confidence interval; NS, not significant; Pc value, P value with Bonferroni correction; Pc value < 0.05 was regarded to have statistical significance; * Fischer's exact test

Two of the 13 tag SNPs detected were found to be significantly associated with VKH disease. Significant eQTL and sQTL signals were captured for *CARD9* rs3812555, which was considered to affect gene regulation (Additional file 1: Table S5). Additionally, bioinformatics data from the HaploReg v4.1 databases suggested the presence of epigenetic regulatory activity at the rs3812555 loci, which may play a critical role in regulating autoimmune disorders, such as VKH diseases. However, to our knowledge, the SNP rs74437127 has not been reported yet. In subsequent experiments, we investigated whether rs3812555 had biological functions.

### LD and haplotype analysis

LD plots and haplotypes of *PRKCD* and *CARD9* were generated on the SHEsis online platform using data from all the subjects in the current study. The Dʹ values for all pairwise SNPs were calculated. Strong LD was noted among three *PRKCD* polymorphisms, that is, rs2306572, rs74437127, and rs45596236 (Additional file 1: Fig. S2A), and the frequency of the *PRKCD* ATG haplotype in patients with VKH was significantly lower than that of controls (Pc = 3.11 × 10^–3^, OR = 0.594, 95% CI = 0.437–0.807; Table 3). Five tag SNPs (rs4073153, rs9411205, rs3812555, rs59902911, and rs11145769) in *CARD9* were also in strong LD (Additional file 1: Fig. S2B) and the frequency of the GCTTA haplotype (order of SNPs: rs4073153, rs9411205, rs3812555, rs59902911, and rs11145769) in patients with VKH was also significantly lower than that of controls (Pc = 5.00 × 10^–3^, OR = 0.639, 95% CI = 0.484–0.843; Table 4).

**Table 3:** Haplotype frequencies of the three tag SNPs within *PRKCD*

Haplotype^a^	Case (freq)^b^	Control (freq)^b^	χ2	Pearson’s P	Pc value	Odds ratio [95%]
A C A	58.74 (0.032)	53.61 (0.031)	0.084	0.772	NS	1.057 [0.726–1.540]
A C G	1295.28 (0.714)	1208.84 (0.693)	2.170	0.141	NS	1.116 [0.964–1.292]
A T G	69.95 (0.039)	110.55 (0.063)	11.306	7.77 × 10^–4^	3.11 × 10^–3^	0.594 [0.437–0.807]
G C A	365.13 (0.201)	350.25 (0.201)	0.004	0.951	NS	1.005 [0.853–1.185]

^a^The sequence of the *PRKCD* tag SNPs (rs2306572, rs74437127, rs45596236)

^b^The frequencies of haplotype were calculated using SHEsis online platform. Haplotypes were selected with minor frequency greater than 0.03

**Table 4:** Haplotype frequencies of the five tag SNPs within *CARD9*

Haplotype^a^	Case (freq)^b^	Control (freq)^b^	χ2	Pearson’s P	Pc value	Odds ratio [95%]
A C C C G	97.89 (0.065)	84.72 (0.060)	0.267	0.605	NS	1.083 [0.801–1.462]
A C C C G	102.96 (0.065)	88.40 (0.060)	0.182	0.670	NS	1.066 [0.794–1.430]
A T C C G	1091.84 (0.689)	961.79 (0.651)	2.015	0.156	NS	1.123 [0.957–1.317]
G C C C G	232.01 (0.146)	206.54 (0.140)	0.071	0.790	NS	1.028 [0.839–1.260]
G C T T A	93.29 (0.059)	128.62 (0.087)	10.175	0.001	0.005	0.639 [0.484–0.843]

^a^ The sequence of the *CARD9* tag SNPs (rs4073153, rs9411205, rs3812555, rs59902911, rs11145769)

^b^The frequencies of haplotype were calculated using SHEsis online platform. Haplotypes were selected with minor frequency greater than 0.03

### Relationship between genotypes and gene expression at the mRNA level

**Fig. 1:**
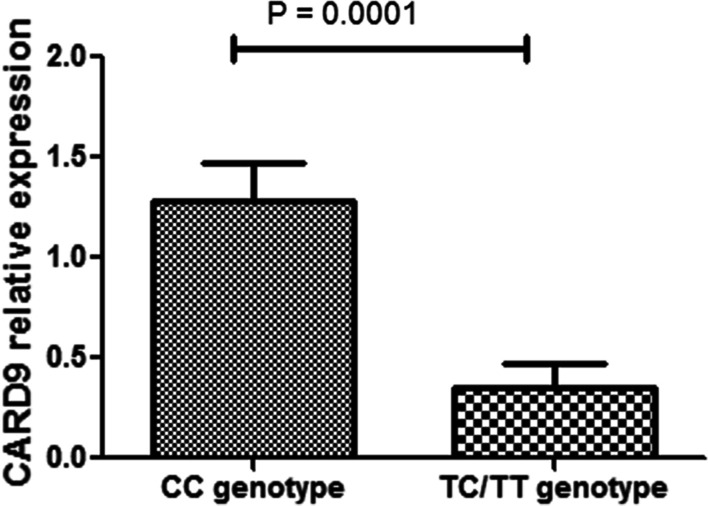
Relationship between rs3812555 and *CARD9* expression. Description: *CARD9* expression in anti-CD3/CD28-stimulated PBMCs from healthy controls with different genotypes of rs3812555 (CC = 22, TC = 15, TT = 1)

As mentioned above, different genotypes of *CARD9* rs3812555 were found to be associated with susceptibility to VKH disease. Therefore, real-time PCR analysis was used to measure the relationship between rs3812555 polymorphism and *CARD9* gene expression in PBMCs under normal or inflammatory conditions in 38 normal controls. We found that the different genotypes of rs3812555 were not significantly associated with *CARD9* expression when PBMCs were unstimulated or stimulated with LPS (P > 0.05; Additional file 1:Fig. S3A, B). After the PBMCs were stimulated with anti-CD3/CD28 antibodies, individuals with the CC genotype of the rs3812555 showed significantly higher *CARD9* gene expression than those with the TC/TT genotype (*P* = 1.00 × 10^–4^; Fig. 1).

### Relationship between genotypes and cytokines expression

**Fig. 2:**
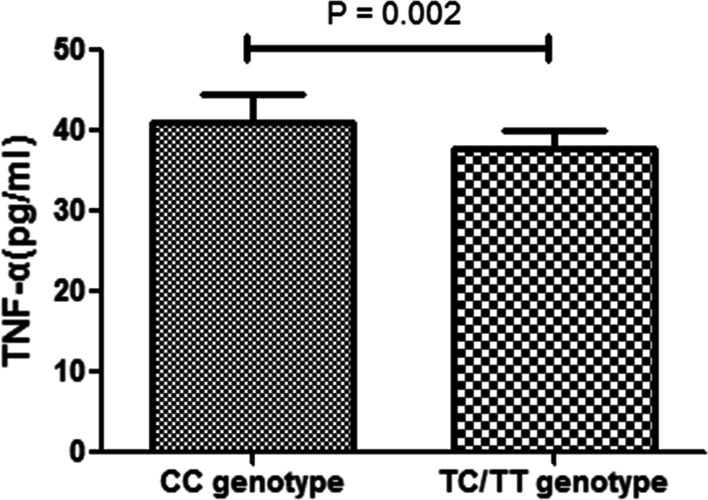
Relationship between rs3812555 and cytokines production. Description: The production of TNF-α by anti-CD3/CD28-stimulated PBMCs from healthy controls with different genotypes of rs3812555 (CC = 22, TC = 15, TT = 1)

We studied whether the rs3812555 polymorphisms could affect cytokine production in PBMCs treated with anti-CD3/CD28 antibodies. ELISA was performed to examine the IL-6, IL-1β, IL-17, TNF-α, and IL-23 levels in 72 h cell culture supernatants. We found that the TNF-α production in CC carriers was significantly higher than that in TC/TT carriers (*P* = 2.00 × 10^–3^; Fig. 2). On analysing the rs3812555 polymorphism data, we found that genotype did not affect IL-1β, IL-6, IL-17, and IL-23 production (P > 0.05; Additional file 1:Fig. S4A–D).

## Discussion

The pathogenesis of VKH disease remains uncertain; however, aberrant activation of the Th1 and Th17 pathways in genetically predisposed individuals has been found to be involved in the development of this disease [21]. The PRKCD–CARD9–nuclear factor (NF)-κB signalling pathway induces maturation of dendritic cells into effector APCs, is involved in secretion of pro-inflammatory cytokines, and directs Th1/Th17 polarisation of T cells [22–24]. In addition, it plays a central role in inducing Th17-related responses and in experimental autoimmune uveitis (EAU) pathogenesis [25, 26]. In the current study, two susceptibility SNPs, rs74437127 and rs3812555, were found to be associated with VKH disease in the Chinese Han population. In addition, we found rs3812555 as a functional variant, wherein individuals with the CC genotype of rs3812555 showed increased *CARD9* gene expression and TNF-α production. Based on previous studies and our results, we hypothesised that abnormal response or dysfunction of CARD9 pathway may occur in VKH patients with rs3812555 CC genotype. In the future, abnormalities in CARD9 signalling pathway may be used to guide the clinical treatment of VKH and the development of related targeted drugs.

To our knowledge, this study is the first to identify that the *PRKCD* rs74437127 C allele is a risk allele and that the T allele is a protective factor for VKH disease. The CC genotype and C allele of *CARD9* rs3812555 were found to increase susceptibility of VKH disease, which were consistent with a study on AS [17]. These indicated that *CARD9* genetic variations might have a similar promoting role in the pathogenesis of AS and VKH disease. Moreover, *CARD9* rs59902911 were found to be associated with RA in European Americans (*P* = 1.01 × 10^–6^) [18]. Nevertheless, in our study, uncorrected p values indicated that individuals carrying the rs59902911 CC genotype and C allele had an increased susceptibility of VKH disease, but the significance was lost after using Bonferroni correction, which was a similar observation to that of an earlier study on Behcet’s disease (BD) [27]. The correlation between SNP rs59902911 and susceptibility to VKH disease should be confirmed in other races in future studies. We also detected associations with haplotypes similar to those of the significant tag SNPs. The T allele of rs74437127 and rs3812555, a protective allele for VKH disease, was found in the protective haplotypes ATG (order of SNPs: rs2306572, rs74437127, and rs45596236) and GCTTA (order of SNPs: rs4073153, rs9411205, rs3812555, rs59902911, and rs11145769), respectively. Similar *CARD9* haplotype results have been reported for BD [27]; however, the haplotype composition differed from that found in our study. The biological functions of the ATG and GCTTA haplotypes described in the current study had not been reported earlier. The mechanism by which these haplotypes affect VKH disease occurrence and development remains unclear and requires further investigation.

Bioinformatics data obtained from the GTEx and HaploReg v4.1 databases indicated that genetic alterations occurring in the SNP rs3812555 may be associated with the alteration of *CARD9* mRNA levels [28, 29]. To prove this assumption, we examined the association of the *CARD9* rs3812555 polymorphism with mRNA and cytokine expression in normal controls but not in patients with VKH, mainly to exclude a confounding effect of the inflammatory response or installed therapy. The functional study showed that rs3812555 CC carriers had significantly greater *CARD9* expression than TC/TT carriers when PBMCs were simulated with anti-CD3/CD28 antibodies. Our findings and those from a previous study showed that activation of the adapter protein CARD9 helped mediate T cell differentiation under inflammatory conditions [24], suggesting that different genotypes of rs3812555 may have different effects on the regulation of T cell effector functions. The result suggests that the rs3812555 CC genotype, which increases the susceptibility of acquiring VKH, may be linked to increased *CARD9* mRNA expression. Previous studies involving patients with other autoimmune diseases reported that *CARD9* mRNA expression increased during active inflammation in patients with IBD [30]. In addition, in a mouse study, *Card-9* mRNA expression was increased in the murine kidney during lupus nephritis progression [31]. These two findings are further supported by our hypothesis. In the next experiment, we investigated five cytokines associated with VKH pathogenesis [32–34], namely IL-6, IL-1β, IL-17, TNF-α, and IL-23, to study the relationship between rs3812555 genotype and cytokine expression. We found that rs3812555 CC carriers had higher TNF-α levels than TC/TT carriers. The result is consistent with that of a study showing that *CARD9* polymorphisms positively regulated TNF-α production [35]. TNF-α is a major pro-inflammatory hallmark cytokine produced downstream of CARD9-activated p38 MAPK [16, 36]; the elevated TNF-α production noted in rs3812555 CC carriers is in agreement with the role played by this genotype in susceptibility to VKH disease. However, in this study, we did not find a significant association between the production of IL-1β, IL-6, IL-17, and IL-23 and two genotypic groups of rs3812555. Taken together, our results suggest that the rs3812555 polymorphism may increase susceptibility to VKH disease by regulation of *CARD9* gene expression and pro-inflammatory cytokine, such as TNF-α, production.

Given that the TNF-α inhibitor adalimumab (ADA) has shown promising efficacy in patients with VKH [37] and our current results that *CARD9* mRNA and TNF-α levels were higher in rs3812555 CC genotype individuals, we propose that patients who have VKH and carry the rs3812555 CC genotype are more likely to obtain better treatment outcomes with ADA than TC/TT genotype carriers.

Our study has several limitations. Firstly, all the participants were Han Chinese; therefore, our conclusions need to be confirmed in diverse ethnicities. Secondly, selection bias may have existed because all the patients with VKH in this study were enrolled from an ophthalmology clinic. Therefore, patients who had VKH and were being treated in other departments should be recruited to confirm the results of the current study. Thirdly, we could not perform functional studies for *PRKCD* rs74437127 because the rs74437127 T allele had low frequency in the Chinese Han population (T = 0.068); genotyping of rs74437127 from the 38 normal individuals showed that the samples were all of the CC genotype. In future studies, we will use larger sample sizes to help elucidate the exact role played by rs74437127 in VKH disease development.

## Conclusions

In conclusion, we not only found an association between the *PRKCD* rs74437127 and *CARD9* rs3812555 polymorphisms and VKH susceptibility, but also revealed that the increased susceptibility of rs3812555 for VKH may be mediated by regulating *CARD9* gene expression and the production of pro-inflammatory cytokines, such as TNF-α. Thus, SNP rs3812555 identified in this study, as a functional variant, may provide new insight into gene therapy for VKH disease in the Chinese Han population.

## Materials and methods

### Subjects

We recruited 912 patients with VKH and 878 normal controls from the ophthalmology clinic of First Affiliated Hospital of Chongqing Medical University (Chongqing, China), all of whom were matched for sex, age, and ethnicity. All the subjects were Han Chinese and voluntarily signed an informed consent form before blood collection. We strictly followed both sets of criteria proposed by the International Research Group and Yang et al. for diagnosing VKH disease [38, 39]. Meanwhile, normal individuals without autoimmune diseases, severe systemic diseases (e.g. hypertension, diabetes, and infectious diseases, etc.), or a family history of VKH disease were selected as controls. The design of this study was based on the principles of the Declaration of Helsinki and approved by the Clinical Research Ethics Committee of the Second Affiliated Hospital of Fujian Medical University (NO.2019–110) and the Ethics Committee of Chongqing Medical University (NO.2009–201,008).

### Selection of tagging single-nucleotide polymorphisms (tag SNPs)

CHB data (Han Chinese in Beijing, China) for *PRKCD* and *CARD9* SNPs were downloaded from the 1000 Genomes Project database (http://grch37.ensembl.org/) and analysed using Haploview 4.2 software. The criteria for selecting tag SNPs included minimum minor allele frequency (MAF) set at 0.05 and linkage disequilibrium (LD) correlation coefficient (r^2^) threshold at 0.8. We identified 13 tag SNPs in *PRKCD*. The primers of the three tag SNPs (rs3773722, rs7634447, and rs11130351 in *PRKCD*) did not pass quality test; therefore, 10 tag SNPs of *PRKCD* were finally chosen. In addition, five tag SNPs captured genetic information on 16 common *CARD9* SNPs [27]. In total, 15 candidate tag SNPs were selected in our study: 10 tag SNPs for *PRKCD* (rs4687706, rs3773732, rs6797662, rs6764111, rs3821689, rs1308486, rs78346230, rs2306572, rs74437127, and rs45596236) and five tag SNPs for *CARD9* (rs4073153, rs9411205, rs3812555, rs59902911, and rs11145769). These tag SNPs cover most of the common allelic variations within the transcribed regions of the *PRKCD* and *CARD9* genes.

### DNA extraction

DNA of participants was extracted from peripheral blood samples with the NPure Blood DNA Kit (BioBase, Chengdu, China) and tested for DNA concentration and purity with an ultraviolet spectrophotometer, as per the manufacturer’s protocols. The DNA sample concentration was defined as ≥ 30 ng/UL, and the A260/A280 ratio of the sample was defined as > 1.8. The qualified DNA was diluted to a concentration of 30–50 ng/μL and stored at − 20 °C until use.

## Genotyping

The MassARRAY Assay design 3.1 software was used to design genotyping primers (Additional file 1: Table S1). Genotyping of the tested tag SNPs was performed using the MassARRAY system (Sequenom, San Diego, CA, USA) and iPLEX Gold Assay. The genotyping data were analysed using the MassARRAY Typer 4.0 software. All procedures were performed as per the manufacturers’ protocols. We use the HaploReg v4.1 (http://www.broadinstitute.org/mammals/haploreg/haploreg.php; in the public domain) [28] and GTEx (http://www.gtexportal.org/home/; in the public domain) databases [29] to examine the potential biological functions of the significant tag SNPs.

### Cell separation and culture

Ficoll-Hypaque density-gradient centrifugation was used to extract peripheral blood mononuclear cells (PBMCs) from whole blood of 38 normal controls. The PBMCs isolated were seeded in 24-well plates (1 × 10^6^ cells/well), and both cultured in the Roswell Park Memorial Institute (RPMI) 1640 standard culture medium containing 10% foetal calf serum (FCS; Greiner, Wemmel, Belgium), penicillin (100 μg/ml), and streptomycin (100 μg/ml). One portion of the PBMCs was subjected to anti-CD3/CD28 antibodies (5:1; Miltenyi Biotec, Palo Alto, CA) for 72 h to simulate antigen presentation. The other cells were cultured in lipopolysaccharide (LPS; 100 ng/mL; Sigma, MO, USA) for 24 h to simulate an inflammatory signal. Cell culture supernatants were collected to detect IL-6, IL-1β, IL-17, TNF-α, and IL-23 concentrations. Cell precipitation was used to detect gene expression levels.

### Real-time quantitative polymerase chain reaction (PCR)

TRIzol (Invitrogen, San Diego, CA) was utilised to extract total RNA from PBMCs, which was reverse transcribed into cDNA with a transcriptase kit; both procedures were performed as per the manufacturers’ instructions. Real-time quantitative PCR was used to analyse *CARD9* expression on an ABI 7500 Software v2.0.6; β-actin was chosen as the internal control gene. The following primers were used for these analyses: β-actin: forward, 5'-GGATGCAGAAGGAGATCACTG-3' and reverse, 5'-CGATCCACACGGAGTACTTG-3'; and *CARD9*: forward, 5'-GCAGGTGTTCCAGTGTGAGG-3' and reverse, 5'-GTGAGCCATCTTCCAGGTCG-3'. The 2^−∆∆CT^ method was performed to detect relative expression levels of *CARD9*.

### Enzyme-linked immunosorbent assay (ELISA)

Human Duoset ELISA development kits (R&D Systems, Minneapolis, MN, USA) were utilised to determine the cytokine levels, including IL-6, IL-1β, IL-17, TNF-α, and IL-23 in the culture supernatants of PBMCs after PBMCs had been cultured with anti-CD3/CD28 antibodies for 72 h.

### Statistical analysis

The tested tag SNPs with a call rate above 90% were considered for statistical analysis [40]. All statistical analyses were performed with SPSS 25.0 software. Chi-square (χ2) test was utilised to analyse Hardy–Weinberg equilibrium (HWE) in normal controls, and two tag SNPs in *PRKCD* (rs13084863 and rs6797662) were excluded from further analysis because of deviation from the HWE. Either χ2 or Fisher’s exact test was applied to detect differences in genotype and allele frequencies of 13 tag SNPs between patients and normal controls. Bonferroni-corrected p value (Pc) was equal to the p value multiplied by 13, and a Pc < 0.05 was considered statistical significance. LD and haplotype analyses were performed using the SHEsis online platform (http://analysis.bio-x.cn/myanalysis.php). The Mann–Whitney *U* test was applied to evaluate *CARD9* expression and cytokine data between different genotypic groups. In addition, statistical power analysis was performed using the GAS power calculator (http://csg.sph.umich.edu/abecasis/gas_power_calculator/).

## Supplementary Information

**Additional file 1**. Supplemental material including Supplemental Tables S1–S5, Supplemental  Figures S1–S3.

## Data Availability

All data generated or analysed during this study are included in this published article and its supplementary information files.

## Abbreviations

*PRKCD*: Protein kinase C delta
*CARD9*: Caspase recruitment domain family member 9
VKH: Vogt–Koyanagi–Harada
IL: Interleukin
TCR: T cell receptor
TNF: Tumour necrosis factor
SLE: Systemic lupus erythematosus
IBD: Inflammatory bowel disease
CD: Crohn’s disease
APC: Antigen-presenting cell
RA: Rheumatoid arthritis
AS: Ankylosing spondylitis
SNP: Single-nucleotide polymorphism
tag SNP: Tagging single-nucleotide polymorphism
MAF: Minor allele frequency
LD: Linkage disequilibrium
r^2^: Correlation coefficient
eQTL: Expression quantitative trait locus
sQLT: Splicing quantitative trait locus
PMBC: Peripheral blood mononuclear cell
PCR: Polymerase chain reaction
ELISA: Enzyme-linked immunosorbent assay
HWE: Hardy–Weinberg equilibrium (HWE)
Pc: Bonferroni-corrected P values
NF-κB: Nuclear factor NF-κB
EAU: Experimental autoimmune uveitis
BD: Behcet’s disease
ADA: Adalimumab
