# Supplementary material for: Genetic association of PRKCD and CARD9 polymorphisms with Vogt–Koyanagi–Harada disease in the Chinese Han population
Source: Hum Genomics. 2023 Feb 13;17:9. doi: 10.1186/s40246-023-00459-7 (PMC9926551; doi:10.1186/s40246-023-00459-7)
Supplement: Supplementary file 1 — Additional file 1. Supplemental material including Supplemental Tables S1–S5, Supplemental Figures S1–S3. [file 40246_2023_459_MOESM1_ESM.docx]

Supplementary Table S1. The genotyping primer sequences of *PRKCD* and *CARD9.*

| **Gene** | **SNPs ID** | **Alleles** | **2nd-PCRP** | **1st-PCRP** | **UEP_SEQ** |
| --- | --- | --- | --- | --- | --- |
| *PRKCD* | rs4687706 | G/A | ACGTTGGATGAGATTGACCAGCTCAAGCAC | ACGTTGGATGTGCTCCATGGCGTGCCTGA | TGGAGCGCAAGCACAC |
| *PRKCD* | rs3773732 | A/G | ACGTTGGATGCCTCCTCATCCACTGTAAAG | ACGTTGGATGCTTTTTGGACGTGGATGTGC | CACTGTAAAGGCCCTCGG |
| *PRKCD* | rs6797662 | C/T | ACGTTGGATGATCCTCAGAGGACAGCTCAC | ACGTTGGATGCCACCCCCTACACTGTTTC | ACAGCTCACCAAACC |
| *PRKCD* | rs6764111 | A/G | ACGTTGGATGTCAGGCCACACTCAGAGCA | ACGTTGGATGGTTTGTGGTTTTCCCTCCAG | AGCTGCTGCTCACATTT |
| *PRKCD* | rs3821689 | T/C | ACGTTGGATGTATGGTGCACATTCATGCCG | ACGTTGGATGCTTTCTCTTGCTCTCCTGCG | CTCAGCCAAGGGCAG |
| *PRKCD* | rs13084863 | C/T | ACGTTGGATGCCGCATTCATGCCTCCCAT | ACGTTGGATGATGCATTCAGCAGCCACCCT | cttcgTGCCTCCCATCTCCCA |
| *PRKCD* | rs78346230 | C/T | ACGTTGGATGATCAGCAGCCCAGAGGTAAG | ACGTTGGATGGTTTCCATTTGTCCCAGAGG | CCAGAGGTAAGGAAAGG |
| *PRKCD* | rs2306572 | A/G | ACGTTGGATGTCTCCAGGCTCCTCCTTCG | ACGTTGGATGTCTACACAAAGCCACCGTTC | GCAGGAGGAAGACTCAAG |
| *PRKCD* | rs74437127 | C/T | ACGTTGGATGAGGTAAGGTGTGATGCGTGA | ACGTTGGATGACTACGAGAACGATGACGAG | gCGGAAGCCCTCCAGGACG |
| *PRKCD* | rs45596236 | G/A | ACGTTGGATGAACCTAACCAAACCCCTCCT | ACGTTGGATGAACCTGCTAGGCTGCCTGAT | GGGGTGAGGGCACAC |
| *CARD9* | rs4073153 | A/G | ACGTTGGATGACATGGACACGCCTCAAAAC | ACGTTGGATGATTTTAAGTGCCTTCCACTG | gTAACAGACTTCTTACACAGA |
| *CARD9* | rs9411205 | C/T | ACGTTGGATGCACGTGTGCTGATGTATACC | ACGTTGGATGATGCATTCCTGTTCCTGCTG | ttATGTATACCAGCACAGGAG |
| *CARD9* | rs3812555 | C/T | ACGTTGGATGAGTTGGACAAGCTTCCTGAG | ACGTTGGATGTTCTGAGCTGCAGCCAAAGG | cggtAAGGAGGAGCCGCAGAC |
| *CARD9* | rs59902911 | C/T | ACGTTGGATGCTCTTGGGATGTTGTTGTGC | ACGTTGGATGTATCACAGGCTGAGGGTCAC | ttgaGGGAGAGCTAGGGGTTG |
| *CARD9* | rs11145769 | G/A | ACGTTGGATGTGTGCTCCTGGACATCCTG | ACGTTGGATGACCTTCTTGTACAGCTGCGG | ggcgtACCGGCCACAAGGGCTA |

Supplemental Table S2. Parameters chosen for statistical power analyses.

| **Parameter** | **Value** | **Note** |
| --- | --- | --- |
| Cases | 912 | Sample size |
| Controls | 878 | Sample size |
| Significance Level | 0.0038 | 0.05/13≈0.0038 |
| Prevalence | 0.00001 | Cao S, et al. Br J Ophthalmol. 2016;100(3):436-442. |
| Disease Allele Frequency | 0.197 | Average MAF of the 13 selected SNPs |
| Genotype Relative Risk | 1.4 | - |

Supplementary Figure S1. Statistical power achieved by the study setting versus genotypic relative risk.


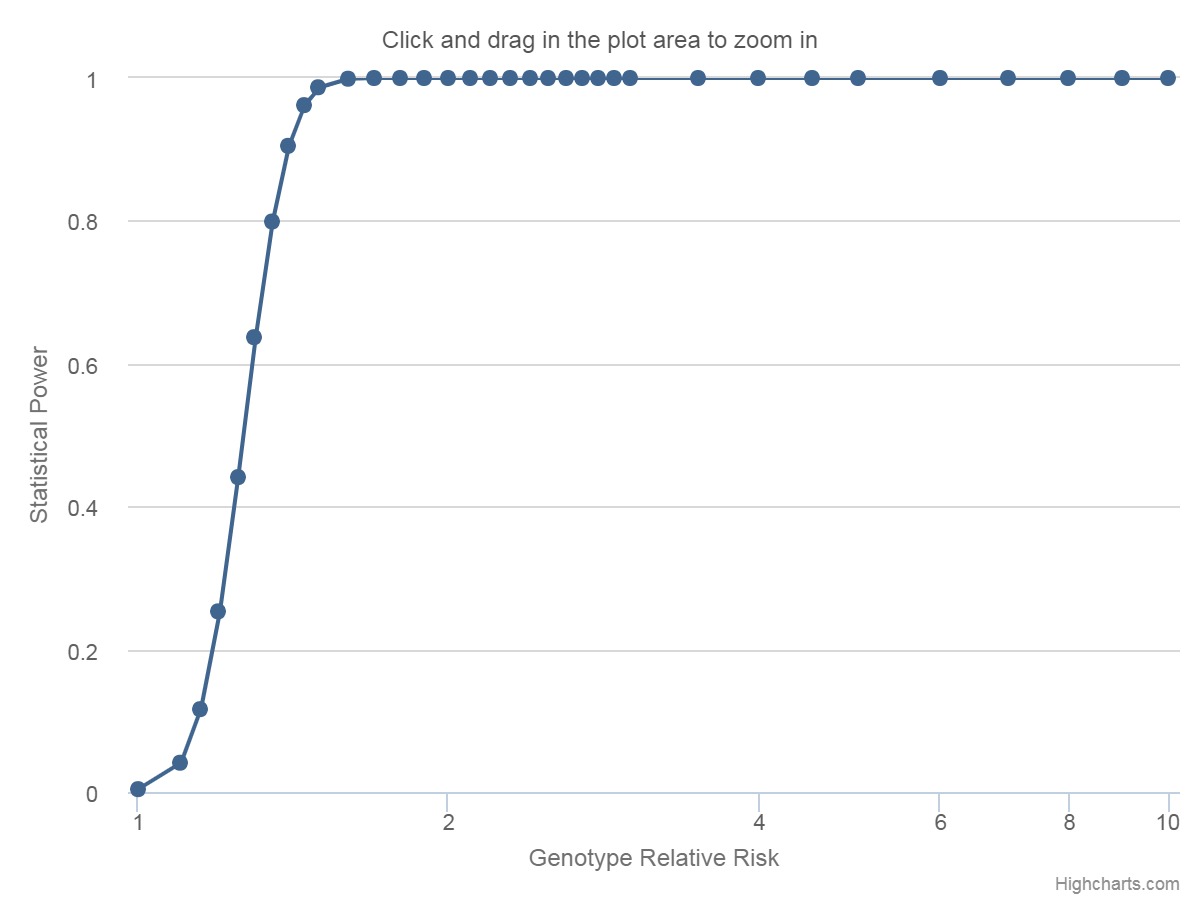


90.5%, RR=1.4

The figure was generated by GAS power calculator (version 4.2.5) http://csg.sph.umich.edu/abecasis/gas_power_calculator/index.html

Supplemental Table S3. Genetic information for the 13 tag SNPs selected for genotyping.

| **CHR** | **Position** | **SNP** | **Gene** | **Function** | **A1** | **A2** | **MAF** | **HWE** | **Call Rate** |
| --- | --- | --- | --- | --- | --- | --- | --- | --- | --- |
| 3 | 53190870 | rs4687706 | *PRKCD* | None | G | A | 0.257 | 0.07 | 98.0% |
| 3 | 53200876 | rs3773732 | *PRKCD* | Intron | A | G | 0.379 | 0.43 | 93.5% |
| 3 | 53207401 | rs6764111 | *PRKCD* | Intron | A | G | 0.058 | 0.54 | 99.9% |
| 3 | 53208917 | rs3821689 | *PRKCD* | Intron | T | C | 0.248 | 0.10 | 97.3% |
| 3 | 53216990 | rs78346230 | *PRKCD* | Intron | T | C | 0.078 | 0.62 | 99.9% |
| 3 | 53217413 | rs2306572 | *PRKCD* | Intron | G | A | 0.204 | 0.14 | 99.6% |
| 3 | 53218837 | rs74437127 | *PRKCD* | Intron | T | C | 0.068 | 0.06 | 99.8% |
| 3 | 53220569 | rs45596236 | *PRKCD* | Intron | A | G | 0.214 | 0.53 | 99.5% |
| 9 | 139259349 | rs4073153 | *CARD9* | Intron | G | A | 0.301 | 0.17 | 94.4% |
| 9 | 139260463 | rs9411205 | *CARD9* | Intron | C | T | 0.364 | 0.11 | 98.5% |
| 9 | 139261933 | rs3812555 | *CARD9* | Intron | T | C | 0.136 | 0.23 | 92.0% |
| 9 | 139265088 | rs59902911 | *CARD9* | Synonymous | T | C | 0.078 | 0.16 | 98.0% |
| 9 | 139265870 | rs11145769 | *CARD9* | Synonymous | A | G | 0.087 | 0.27 | 99.7% |

CHR:chromosome; A1: minor allele; A2: major allele; MAF: minor allele frequency; HWE: P-value for Hardy Weinberg equilibrium tests conducted in controls.

Supplementary Table S4. Genotypic and allelic distributions of tag SNPs of *PRKCD* and *CARD9*.

| **Gene** | **SNPs** | **Genotype**  **Allele** | **VKH n (%)** | **Control n (%)** | **P Value** | **Pc Value** | **OR (95%CI)** |
| --- | --- | --- | --- | --- | --- | --- | --- |
| *PRKCD* | rs4687706 | AA | 502(0.562) | 468(0.548) | 0.570 | NS | 1.056(0.875-1.276) |
|  |  | GA | 343(0.384) | 342(0.400) | 0.472 | NS | 0.932(0.769-1.129) |
|  |  | GG | 49(0.055) | 44(0.052) | 0.760 | NS | 1.068(0.703-1.622) |
|  |  | A | 1347(0.753) | 1278(0.748) | 0.727 | NS | 1.028(0.882-1.198) |
|  |  | G | 441(0.247) | 430(0.252) | 0.727 | NS | 0.973(0.835-1.134) |
|  |  |  |  |  |  |  |  |
|  | rs3773732 | AA | 92(0.106) | 100(0.122) | 0.305 | NS | 0.854(0.632-1.154) |
|  |  | GA | 412(0.477) | 357(0.437) | 0.101 | NS | 1.174(0.969-1.423) |
|  |  | GG | 360(0.417) | 360(0.441) | 0.321 | NS | 0.907(0.747-1.100) |
|  |  | A | 596(0.345) | 557(0.341) | 0.806 | NS | 1.018(0.883-1.174) |
|  |  | G | 1132(0.655) | 1077(0.659) | 0.806 | NS | 0.982(0.852-1.133) |
|  |  |  |  |  |  |  |  |
|  | rs6764111 | AA | 4(0.004) | 2(0.002) | 0.687* | NS | 1.925(0.352-10.53) |
|  |  | GA | 50(0.055) | 64(0.073) | 0.115 | NS | 0.736(0.502-1.078) |
|  |  | GG | 858(0.941) | 810(0.925) | 0.173 | NS | 1.295(0.892-1.879) |
|  |  | A | 58(0.032) | 68(0.039) | 0.255 | NS | 0.813(0.569-1.162) |
|  |  | G | 1766(0.968) | 1684(0.961) | 0.255 | NS | 1.230(0.861-1.756) |
|  |  |  |  |  |  |  |  |
|  | rs3821689 | CC | 496(0.551) | 463(0.551) | 0.981 | NS | 1.002(0.830-1.211) |
|  |  | CT | 352(0.391) | 334(0.397) | 0.797 | NS | 0.975(0.804-1.182) |
|  |  | TT | 52(0.058) | 44(0.052) | 0.618 | NS | 1.111(0.735-1.679) |
|  |  | C | 1344(0.747) | 1260(0.749) | 0.868 | NS | 0.987(0.847-1.150) |
|  |  | T | 456(0.253) | 422(0.251) | 0.868 | NS | 1.013(0.869-1.181) |
|  |  |  |  |  |  |  |  |
|  | rs78346230 | CC | 733(0.804) | 721(0.822) | 0.319 | NS | 0.886(0.698-1.124) |
|  |  | CT | 165(0.181) | 147(0.168) | 0.458 | NS | 1.097(0.859-1.401) |
|  |  | TT | 14(0.015) | 9(0.010) | 0.404* | NS | 1.504(0.647-3.492) |
|  |  | C | 1631(0.894) | 1589(0.906) | 0.242 | NS | 0.878(0.705-1.092) |
|  |  | T | 193(0.106) | 165(0.094) | 0.242 | NS | 1.140(0.915-1.419) |
|  |  |  |  |  |  |  |  |
|  | rs2306572 | AA | 563(0.619) | 547(0.624) | 0.825 | NS | 0.979(0.808-1.185) |
|  |  | AG | 300(0.330) | 281(0.321) | 0.676 | NS | 1.043(0.856-1.272) |
|  |  | GG | 46(0.051) | 48(0.055) | 0.692 | NS | 0.919(0.607-1.393) |
|  |  | A | 1426(0.784) | 1375(0.785) | 0.975 | NS | 0.997(0.850-1.170) |
|  |  | G | 392(0.216) | 377(0.215) | 0.975 | NS | 1.003(0.855-1.176) |
|  |  |  |  |  |  |  |  |
|  | rs45596236 | AA | 52(0.057) | 50(0.057) | 0.996 | NS | 1.001(0.671-1.493) |
|  |  | AG | 320(0.352) | 304(0.348) | 0.839 | NS | 1.020(0.840-1.240) |
|  |  | GG | 536(0.590) | 520(0.595) | 0.841 | NS | 0.981(0.812-1.185) |
|  |  | A | 424(0.233) | 404(0.231) | 0.868 | NS | 1.013(0.867-1.184) |
|  |  | G | 1392(0.767) | 1344(0.769) | 0.868 | NS | 0.987(0.845-1.153) |
|  |  |  |  |  |  |  |  |
| *CARD9* | rs4073153 | AA | 487(0.555) | 423(0.519) | 0.135 | NS | 1.157(0.956-1.401) |
|  |  | AG | 309(0.352) | 317(0.389) | 0.119 | NS | 0.855(0.701-1.041) |
|  |  | GG | 81(0.092) | 75(0.092) | 0.981 | NS | 1.004(0.722-1.396) |
|  |  | A | 1283(0.731) | 1163(0.713) | 0.243 | NS | 1.094(0.941-1.272) |
|  |  | G | 471(0.269) | 467(0.287) | 0.243 | NS | 0.914(0.786-1.063) |
|  |  |  |  |  |  |  |  |
|  | rs9411205 | CC | 117(0.130) | 119(0.138) | 0.640 | NS | 0.937(0.712-1.232) |
|  |  | CT | 361(0.401) | 374(0.432) | 0.183 | NS | 0.879(0.728-1.063) |
|  |  | TT | 422(0.469) | 372(0.430) | 0.101 | NS | 1.170(0.970-1.412) |
|  |  | C | 595(0.331) | 612(0.354) | 0.146 | NS | 0.902(0.785-1.037) |
|  |  | T | 1205(0.669) | 1118(0.646) | 0.146 | NS | 1.109(0.965-1.274) |
|  |  |  |  |  |  |  |  |
|  | rs59902911 | CC | 772(0.854) | 701(0.817) | 0.036 | NS | 1.310(1.017-1.687) |
|  |  | TC | 126(0.139) | 145(0.169) | 0.085 | NS | 0.796(0.614-1.032) |
|  |  | TT | 6(0.007) | 12(0.014) | 0.156* | NS | 0.471(0.176-1.261) |
|  |  | C | 1670(0.924) | 1547(0.902) | 0.020 | NS | 1.322(1.045-1.673) |
|  |  | T | 138(0.076) | 169(0.098) | 0.020 | NS | 0.756(0.598-0.957) |
|  |  |  |  |  |  |  |  |
|  | rs11145769 | AA | 9(0.010) | 14(0.016) | 0.297* | NS | 0.615(0.265-1.428) |
|  |  | GA | 139(0.153) | 166(0.190) | 0.039 | NS | 0.771(0.602-0.987) |
|  |  | GG | 761(0.837) | 695(0.794) | 0.019 | NS | 1.332(1.047-1.694) |
|  |  | A | 157(0.086) | 194(0.111) | 0.014 | NS | 0.758(0.608-0.946) |
|  |  | G | 1661(0.914) | 1556(0.889) | 0.014 | NS | 1.319(1.057-1.646) |

VKH, VKH disease; OR, odds ratio; 95 % CI, 95 % confidence interval; NS, not significant;

Pc value, P value with Bonferroni correction; Pc value < 0.05 was regarded to have statistical significance;

* Fischer's exact test; Values with statistical significance are in boldface.

Supplementary Table S5. Full lists of eQTL and sQTL signals identified for SNP rs3812555 on *CARD9*.

| Signals | Gene | SNP | Variant ID | P Value | NES | Tissue |
| --- | --- | --- | --- | --- | --- | --- |
| eQTL | *CARD9* | rs3812555 | chr9_136367481_C_T_b38 | 2.00×10^-20^ | 0.2 | Whole Blood |
| eQTL | *CARD9* | rs3812555 | chr9_136367481_C_T_b38 | 1.10×10^-8^ | 0.14 | Lung |
| eQTL | *CARD9* | rs3812555 | chr9_136367481_C_T_b38 | 1.60×10^-6^ | -0.25 | Cells-Cultured fibroblasts |
| sQTL | *CARD9* | rs3812555 | chr9_136367481_C_T_b38 | 6.30×10^-21^ | 0.54 | Whole Blood |
| sQTL | *CARD9* | rs3812555 | chr9_136367481_C_T_b38 | 6.80×10^-10^ | 0.42 | Lung |

NES, normalized effect size.

Supplementary Figures S2A,B. Linkage disequilibrium plot for *PRKCD* and *CARD9* gene*.*


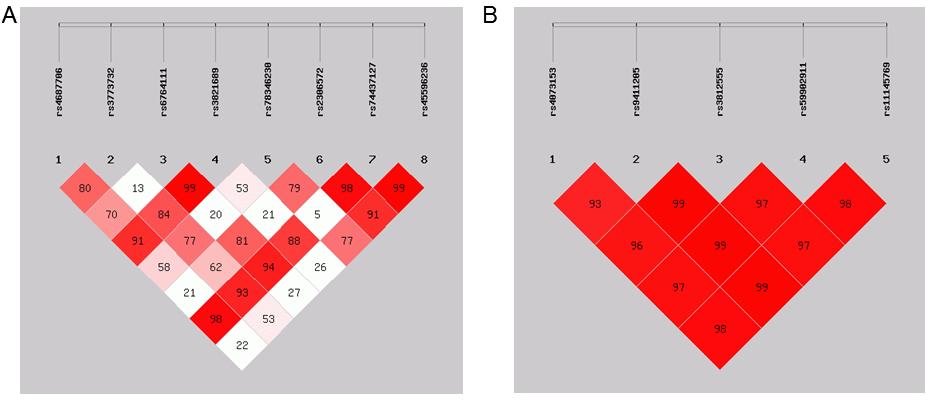


1. The LD analysis of the examined eight tag SNPs of *PRKCD* were generated by SHEsis online platform. Numbers listed in each square represent the Dʹ value for pairwise analysis. (B) The LD analysis of the examined five tag SNPs of *CARD9* were generated by SHEsis online platform. Numbers listed in each square represent the Dʹ value for pairwise analysis.

Supplementary Figures S3A,B. Relationship between rs3812555 and *CARD9* expression.


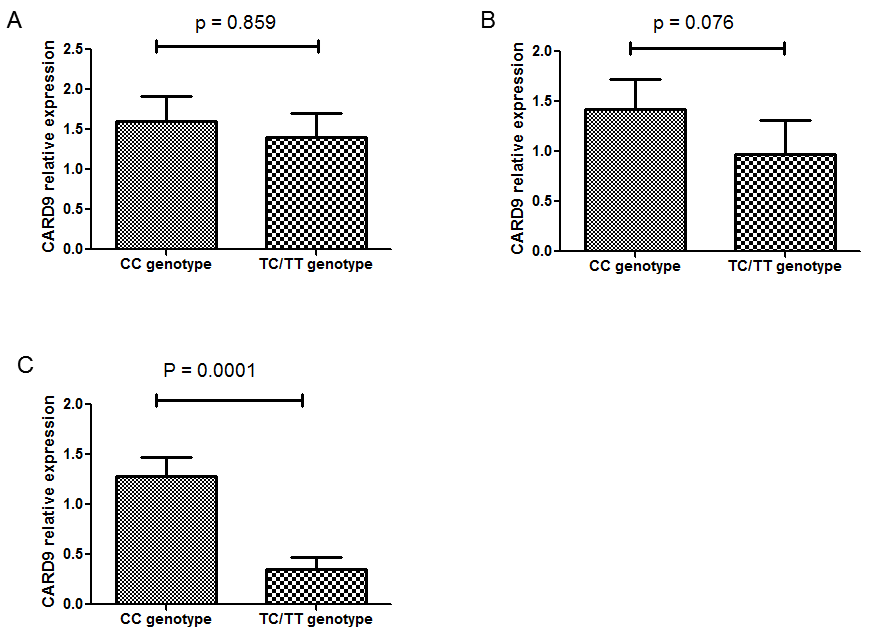


(A) *CARD9* expression in unstimulated PBMCs from healthy controls with different genotypes of rs3812555 (CC = 22, TC = 15, TT = 1). (B) *CARD9* expression in LPS-stimulated PBMCs from healthy controls with different genotypes of rs3812555 (CC = 22, TC = 15, TT = 1).

Supplementary Figures S4A-D. Relationship between rs3812555 and cytokines production.


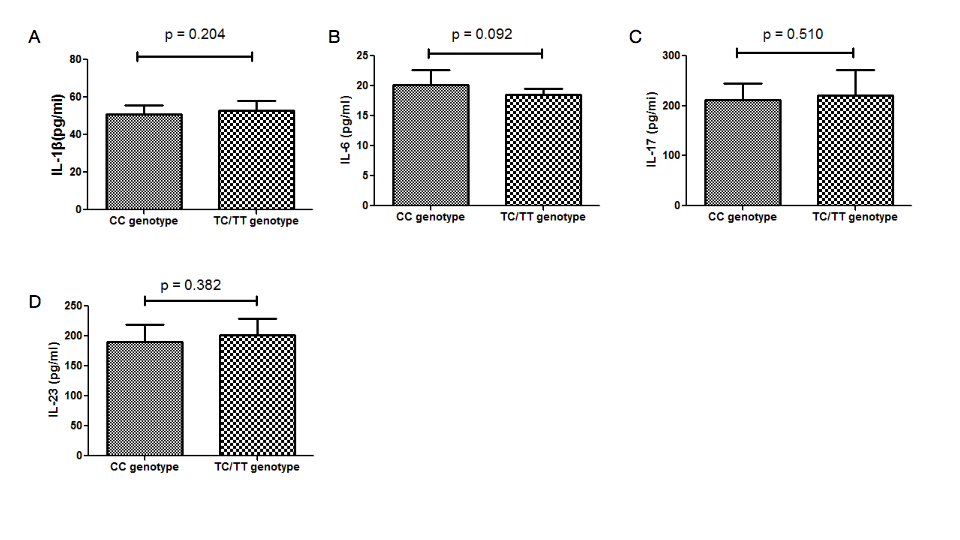


The production of IL-1β (A), IL-6 (B), IL-17 (C), and IL-23 (D) by anti-CD3/CD28-stimulated PBMCs from healthy controls with different genotypes of rs3812555 (CC = 22, TC = 15, TT = 1).
